# Chicken intestinal organoids: a novel method to measure the mode of action of feed additives

**DOI:** 10.3389/fimmu.2024.1368545

**Published:** 2024-05-21

**Authors:** Jordan Mitchell, Kate Sutton, Jeyashree Nathan Elango, Dominika Borowska, Famatta Perry, Ludovic Lahaye, Elizabeth Santin, Ryan J. Arsenault, Lonneke Vervelde

**Affiliations:** ^1^ Division of Immunology, The Roslin Institute and Royal (Dick) School of Veterinary Sciences (R(D)SVS), University of Edinburgh, Edinburgh, United Kingdom; ^2^ Department of Biological Sciences, University of Delaware, Newark, DE, United States; ^3^ Department of Animal and Food Sciences, University of Delaware, Newark, DE, United States; ^4^ Jefo Nutrition Inc., Saint-Hyacinthe, QC, Canada

**Keywords:** organoid, chicken, feed additives, *in vitro*, innate immunity, immunometabolomics, *Salmonella*

## Abstract

There is a rapidly growing interest in how the avian intestine is affected by dietary components and feed additives. The paucity of physiologically relevant models has limited research in this field of poultry gut health and led to an over-reliance on the use of live birds for experiments. The development of complex 3D intestinal organoids or “mini-guts” has created ample opportunities for poultry research in this field. A major advantage of the floating chicken intestinal organoids is the combination of a complex cell system with an easily accessible apical-out orientation grown in a simple culture medium without an extracellular matrix. The objective was to investigate the impact of a commercial proprietary blend of organic acids and essential oils (OA+EO) on the innate immune responses and kinome of chicken intestinal organoids in a *Salmonella* challenge model. To mimic the *in vivo* prolonged exposure of the intestine to the product, the intestinal organoids were treated for 2 days with 0.5 or 0.25 mg/mL OA+EO and either uninfected or infected with *Salmonella* and bacterial load in the organoids was quantified at 3 hours post infection. The bacteria were also treated with OA+EO for 1 day prior to challenge of the organoids to mimic intestinal exposure. The treatment of the organoids with OA+EO resulted in a significant decrease in the bacterial load compared to untreated infected organoids. The expression of 88 innate immune genes was investigated using a high throughput qPCR array, measuring the expression of 88 innate immune genes. *Salmonella* invasion of the untreated intestinal organoids resulted in a significant increase in the expression of inflammatory cytokine and chemokines as well as genes involved in intracellular signaling. In contrast, when the organoids were treated with OA+EO and challenged with *Salmonella*, the inflammatory responses were significantly downregulated. The kinome array data suggested decreased phosphorylation elicited by the OA+EO with *Salmonella* in agreement with the gene expression data sets. This study demonstrates that the *in vitro* chicken intestinal organoids are a new tool to measure the effect of the feed additives in a bacterial challenge model by measuring innate immune and protein kinases responses.

## 1 Introduction

Avian gastrointestinal studies have long been hampered by a lack of representative cell culture tools such as cell lines, but the global movement to reduce experimental animals has resulted in the development of alternative comprehensive lab models that closely resemble the chicken intestinal tract. Since the initial landmark paper by Sato et al. (1) which described the first stem cell-derived 3D intestinal organoid that differentiated into villus-crypt structures that encompassed key epithelial cell lineages found *in vitro*, various progressive models were developed over the past 10 years along with “intestine-on-a-chip” microfluidic bioengineered models for human and mice [reviewed in (2)]. The development of livestock intestinal organoids is progressing although the application of livestock organoids to investigate pharmaceutical and neutraceutical components is lacking compared to the application of human organoids to investigate functional foods (reviewed in (3)). Various chicken organoid models have been described, ranging from enterospheres or spheroids (4) to extracellular matrix (ECM) embedded organoids using mammalian culture methods. These organoids form a central functional lumen lined by highly polarized epithelial cells whose apical brush borders face internally and basolateral surfaces lie in contact with the ECM scaffold (5, 6). A practical limitation of the “basal-out” 3D geometry is that it prevents easy access to the apical surface of the epithelium. The omission of the ECM and additional niche growth factors has led to the development of organoids with an “apical-out” orientation with easy access to the apical epithelium which makes their application more practical and cheaper (7). Although they cannot be passaged like the classical organoids (8) the costs are lower due to a lack of animal-derived products used for classical organoid cultures (ECM), the option to cryopreserve the “apical-out” chicken organoids enables large scale studies and biobanking. A major advantage of the “apical-out” 3D organoids is that they naturally contain all cells of the intestinal epithelium as wells as the underlying lamina propria (7). This complex cell system mimics the cross-talk between epithelial and lamina propria cells which maintains the homeostatic state of the intestine (7, 9). Organoids comprised of only an epithelial cell layer lack the regulatory circuits that are switched on after inflammatory insults (10). These advantages will enable further investigations into host-pathogen interaction and into the mode of action of feed additives.

There is growing interest in the use of feed additives to reduce the use of antibiotic growth promotors (AGP) to improve gut health. When combined with improved biosecurity practices, natural feed additives, such as a combination of organic acids (OA) and essential oils (EO) with proven positive effects on chickens’ intestinal health (11, 12), can play a key role in improving growth performance. Organic acids can be supplied via the feeds but can also originate from endogenous microbial fermentation and some can be naturally found in the intestinal tract of animals, whereas EO blends are mixtures of phytochemical compounds. The main mode of action linked to the synergic effect of OA and EO has been suggested to be via the modulation of the intestinal microbiota, promotion of nutrient absorption and anti-oxidant effects, while antimicrobial properties have also been documented (reviewed in 13–15).

The chicken-specific kinome peptide array technique is designed to measure and evaluate the immunometabolic signaling changes that occur between treatment and control groups (16). The technology utilizes 15 amino acids (AA) long peptides from the chicken proteome corresponding to known kinase target sites in the human proteome (orthologues sequences). Kinases are enzymes which catalyze phosphorylation events, the transfer of a phosphate group from ATP to a target protein, and act as key regulators of cell signaling. This post-translational modification can act to increase or inhibit the target protein’s activity and modulate its capacity to interact with other molecules. Proteins also can contain multiple kinase target sites, sometimes with complementary or enhancing functions and sometimes with antagonistic or competing functions. In the peptide array assay, active kinases in the samples phosphorylate their target sites represented on the array. By considering a proteomic perspective, specifically the post-translational modification of a protein that alters activity, it is easier to generate and interpret phenotypically relevant immune and metabolically integrated data.

Although *in vitro* models are not able to replace performance studies, they can disentangle the effects of feed additives on epithelial barrier integrity and immune status as well as quantify the effect on bacterial invasion. The objective of this study was to validate the 3D chicken intestinal organoids as an *in vitro* tool to measure the effect of feed additives on the potential to improve resilience to microbial challenges and to investigate their mode of action.

## 2 Materials and methods

### 2.1 Generation of 3D intestinal organoids

Experiments were performed using 18–19-day old Hy-Line Brown embryos (*Gallus gallus*) obtained from the National Avian Research Facility, Edinburgh, UK. Embryos were humanely culled under the authority of UK Home Office Project Licences (PE263A4FA) following the guidelines and regulations of the UK Home Office ‘Animals (Scientific Procedures) Act’ 1986. The small intestine (duodenum, jejunum and ileum) was removed, cut open longitudinally then into 3 mm sections and collected in Mg^2+^ and Ca^2+^ free phosphate buffer saline (PBS). For each independent batch, the intestines from five embryos were pooled and a total of eight independent batches were tested. The villi were released from the tissue as previously described (7). In brief, the tissues were digested with *Clostridium histolyticum* type IA collagenase (0.2 mg/mL, Merck, Gillingham, UK) at 37°C for 50 min with agitation at 200 rpm. Single cells were removed by filtering the digestion solution through a 70 µM cell strainer (Corning, Loughborough, UK). The villi were collected by washing the inverted strainer. Villi were collected and pelleted at 100 g for 4 min. The villi were resuspended in Floating Organoid Media (FOM media; Advanced DMEM/F12 supplemented with 1X B27 Plus, 10 mM HEPES, 2 mM L-Glutamine and 50 U/mL Penicillin/Streptomycin; ThermoFisher Scientific, Paisley UK (TFS)) and seeded at ~3000 organoids/well in 6 well plates and incubated overnight at 37°C, 5% CO_2_.

### 2.2 Bacterial strain and culture conditions

*Salmonella enterica* serovar Typhimurium strain 4/74 (STm) was engineered previously to constitutively express GFP by transformation with a derivative of pFVP25·1 (17). Bacteria were cultured in Luria-Bertani broth supplemented with 50 µg/mL of ampicillin (Merck) and 20 µg/mL of naladixic acid (TFS) and incubated for 18 h at 37°C with shaking at 180 rpm. Bacteria were grown to the optical density of 1 at 600 nm and pelleted at 3220 g for 10 min at 4°C. Bacteria were washed twice with PBS and resuspended in 10 mL of antibiotic-free FOM. Tenfold serial dilutions were plated on naladixic acid containing LB agar in duplicate and incubated at 37°C overnight to determine colony forming units (CFU) and expression of GFP was checked under blue light. A titration of the challenge dose was performed using 1000, 500, 250 and 125 CFU of STm per organoid. Infection of organoids for 3 h with 500 CFU or more of STm provided the most reproducible CFU measurements based on CFU counts from homogenized organoids (data not shown). Therefore, 500 CFU of STm was used as the challenge dose in this study.

### 2.3 Treatment of organoids and STm with OA+EO

A proprietary blend of organic acids and essential oils (OA+EO) (Jefo Nutrition Inc. Canada) was prepared at a concentration of 1 mg/mL in antibiotic-free FOM and dissolved at 37°C with regular inversion. After 24 h in culture the organoids were collected by centrifugation at 100 g for 4 min and resuspended in antibiotic-free FOM media or antibiotic-free FOM media supplemented with 0.5 mg/mL (high dose) or 0.25 mg/mL (low dose) of OA+EO in 6 well plates at 37°C, 5% CO_2_. After 24 h, the untreated and the OA+EO treated organoids were collected by centrifugation at 100 g for 4 min and reseeded at 200 organoids per well on 24 well plates in a final volume of 350 µL of antibiotic-free FOM media or antibiotic-free FOM media supplemented with 0.5 mg/mL or 0.25 mg/mL of OA+EO for 24 h at 37°C, 5% CO_2_. The treated organoids were exposed to OA+EO a total of 48 h before inoculation with STm.

STm was resuspended with antibiotic-free FOM media supplemented with high (0.5 mg/mL) or low (0.25 mg/mL) OA+EO and incubated at 4°C for 24 h before inoculation of the organoids.

### 2.4 Infection of organoids with STm and net replication

For bacterial invasion assays, control organoids were infected with 500 CFU/organoid of STm not pre-treated with OA+EO or remained uninfected. Organoids treated for 48 h with OA+EO were infected with 500 CFU/organoid of OA+EO treated STm. To encourage bacterial:organoid interaction, plates were centrifuged for 5 min at 10 g and subsequently incubated at 37°C, 5% CO_2_ for 3 h. For each batch of organoids, 3 wells on a 24 well plate were prepared for bacterial enumeration, 3 wells for RNA isolation, and 3 wells for protein isolation. The experiments were repeated twice with 4 independent batches of organoids in each experiment (total N=8 independent batches).

To quantify the number of bacteria that invaded the untreated and OA+OE treated organoids, organoids were treated with gentamycin (Gibco, 50 μg/mL) for 30 min at 37°C. Three wells were pooled, and centrifuged at 400 g for 4 min. Organoids were washed twice in PBS and homogenized in 300 μL PBS using steel beads in a Tissue-Lyser at 25 Hz for 1 min. Ten-fold serial dilutions were plated on naladixic acid (20 µg/mL) containing LB agar in duplicate and incubated at 37°C overnight to enumerate intracellular bacteria. All input STm inocula were plated out on the day of infection to measure the direct effect of OA+EO on *Salmonella* invasion. The mean and standard deviation were calculated and significant differences between groups were determined by the nonparametric Mann-Whitney U test, with a *P-*value of <0.05 considered statistically significant.

### 2.5 RNA isolation and reverse transcription

Organoids were collected from 3 wells of each treatment group and centrifuged at 2300 g for 4 min. The pellets were lysed in 350 µL RLT Plus buffer with 2β-mercaptoethanol. Total RNA was extracted using a RNeasy Plus Mini Kit (Qiagen) consisting of a genomic DNA column eliminator according to the manufacturer’s instructions and quantified spectrophotometrically. Twenty-five ng/µl of RNA was reversely transcribed using a High-Capacity Reverse Transcription kit (Life Technologies, Paisley, UK), according to the manufacturer’s instructions, with a random hexamer primer and oligo(dT). The cDNA was stored at −20°C until future use.

### 2.6 High-throughput qPCR 96.96 IFC dynamic array

Pre-amplification of cDNA was performed as previously described (18) and unincorporated primers were digested from the pre-amplification samples using 16 U/μl Exonuclease I (*E. coli*, New England Biolabs) at 37°C for 30 min. High-throughput qPCR was performed using 96x96 Integrated Fluid Circuits (IFC) arrays (Standard BioTools) as previously described (19). Samples were amplified in duplicate reactions using primers for 88 genes of interest and seven reference genes. Quantitative PCR was performed on the BioMark HD instrument (Fluidigm) using the thermal cycling conditions as previously described (19). The fluorescence emission was recorded after each cycling step. Raw qPCR data quality threshold was set to 0.65-baseline correction to linear (derivative) and quantitation cycle (Cq) threshold method to auto (global) using the Real-Time PCR Analysis software 3.1.3 (Standard BioTools).

### 2.7 Data processing and analysis

The raw Cq values were processed with GenEx.v6 MultiD Analyses AB, with correction for primer efficiency. *IL1R2* gene was removed from the analysis due to missing over 50% of the data. The stability of the expression of seven putative reference genes - TATA box binding protein (*TBP*), Tubulin alpha chain (*TUBA8B*), beta-actin (*ACTB*), beta-glucuronidase (*GUSB*), glyceraldehyde-3-phosphate dehydrogenase (*GAPDH*), Beta-2-Microglobulin (*B2M*) and ribosomal 28S (*r28S*) - was evaluated via the NormFinder tool in GenEx. The geometric mean of the most stable genes (*ACTB, B2M, TBP*) was used to normalize all samples. Technical replicates were averaged, and the relative quantification values were assessed to the maximum Cq value obtained per gene, transformed to the logarithmic scale.

Statistical analysis of the gene expression of organoids treated with OA+EO and STm from the IFC array was conducted to identify significantly differentially expressed genes (DEGs) between stimulated groups and was performed using GenEx6, with group means compared with two-way t-tests adjusted for multiple comparisons with *post hoc* Bonferroni correction, with significant DEGs having a fold change >1.5 and <−1.5, illustrated in heat maps, and the shared or unique genes annotated in Venn diagrams. For all statistical analyses, *P*-values < 0.05 were considered significant. All statistical analyses were conducted using GraphPad Prism 9 or GenEx v6.

### 2.8 Kinome peptide array

Organoids were centrifuged at 2300 g for 4 min and the pellet was snap-frozen in liquid nitrogen and stored at -80°C. Samples were shipped on dry ice to the University of Delaware, for kinome peptide array analysis.

The kinome peptide array was performed as described by (20). Forty mg of samples were lysed using bead-based homogenization in 100 μL of lysis buffer containing protease and phosphatase inhibitors. The lysed organoids were incubated pelleted at 14,000 *g* for 10 min at 4°C. An aliquot of supernatant was mixed with 10 μL of activation mix containing ATP as the phosphate group donor. Eighty μL of the supernatant-activation solution was applied to the peptide microarray. The custom-designed peptide arrays were obtained from JPT Peptide Technologies (Berlin, Germany), based on in-house sequence designs. A 25 × 60 mm, glass lifter slip was then applied to the microarray to sandwich and disperse the applied lysate.

The microarrays were incubated in a humidity chamber at 40°C and 5% CO_2_. Arrays were placed in a 50 mL centrifuge tube containing PBS-1% Triton, to remove the lifter slip from the microarray surface, and submerged in 2M NaCl-1% Triton and agitated for a minimum of 30 s. This process was repeated with fresh 2M NaCl-1% Triton and arrays were washed in double distilled water with agitation.

Array slides were submerged in phosphospecific fluorescent ProQ Diamond Phosphoprotein Stain (Life Technologies, Carlsbad, CA) on a shaker table at 50 rpm for 1 h and destained twice for 10 min with agitation at 50 rpm in 20% acetonitrile (EMD Millipore Chemicals, Billerica, MA) and 50 mM sodium acetate (Sigma Aldrich, St. Louis, MO). The arrays were then washed with double distilled water, spun dried, and scanned using a Tecan PowerScanner microarray scanner (Tecan Systems, San Jose, CA) at 532 to 560 nm with a 580 nm filter to detect dye fluorescence.

### 2.9 Kinome peptide array data analysis

Images were gridded using GenePix Pro software, and the spot intensity signal was collected as the mean of pixel intensity using local feature background intensity calculation with the default scanner saturation level. The resultant data was analyzed by the PIIKA2 peptide array analysis software (http://saphire.usask.ca/saphire/piika/index.html) (21). Briefly, the resulting data points were normalized to eliminate variance due to technical variation, for example, random variation in staining intensity between arrays or between array blocks within an array. Variance stabilization normalization was performed. Using the normalized data set comparisons between treatment and control groups were performed, calculating fold change and a significance *P*-value. The *P*-value was calculated by conducting a one-sided paired *t*-test between treatment and control values for a given peptide. The resultant fold change and significance values were then used to generate higher order analysis (heat maps, hierarchical clustering, principal component analysis, pathway analysis, etc.).

As described by Perry et al. (22), post PIIKA2 analysis was performed using the following online databases and tools; STRING database (23) Kyoto Encyclopedia of Genes and Genomes (KEGG) pathways and KEGG color and search pathways (24), PhosphoSitePlus (25), Uniprot (26), and Venny 2.1 (27).

## 3 Results

### 3.1 OA+OE reduced *Salmonella* invasion of chicken 3D intestinal organoids

To investigate the effect of OA+EO on *Salmonella enterica* serovar Typhimurium (STm) invasion and its effects on the innate immune responses, we mimicked the *in vivo* circumstances by pre-treating the organoids for 48 h and STm with OA+EO for 24 h prior to infection similar to expose in the intestinal tract. The treatment of organoids with low (0.25 mg/mL) or high (0.5 mg/mL) OA+EO did not alter the morphology of the organoids compared to the untreated organoids (**Supplementary Figure 1**). The effect of OA+EO on the viability of STm has been described previously (14) and in our study, the OA+EO treatment of STm for 24 h at 4°C also reduced the viability, on average by 28%, compared to storage in a floating organoid medium (FOM) for 24 h at 4°C (data not shown).

**Figure 1:**
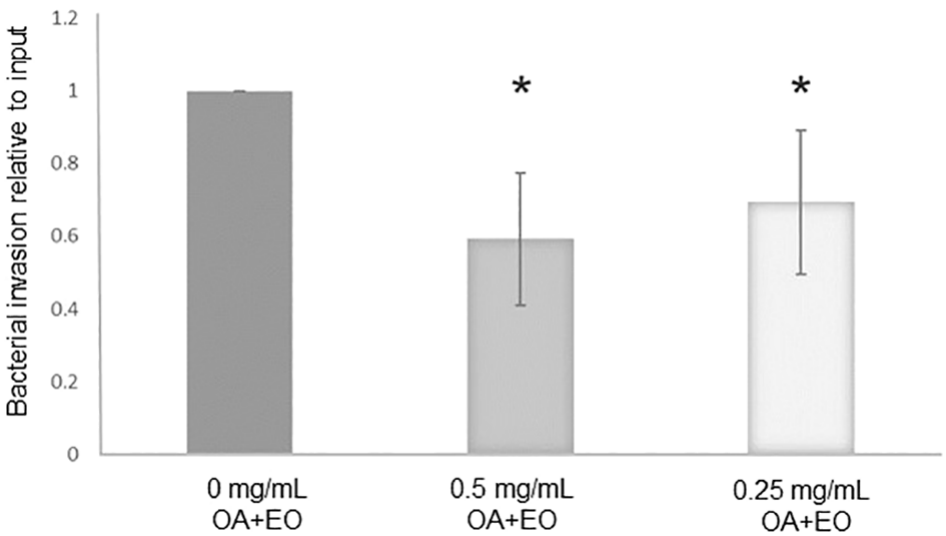
Treatment of chicken 3D organoids with OA+EO reduces *Salmonella* Typhimurum (STm) invasion. Organoids were treated for 48 h with a blend of organic acids and essential oils (OA+EO) at a concentration of 0, 0.5 or 0.25 mg/mL followed by infection with 500 CFU/enteroid of STm. STm was treated for 24 h at 4°C with the respective concentration of OA+EO or remained untreated. After 3 h the extracellular bacteria were killed using gentamycin and cells were harvested, lysed and bacterial invasion enumerated. The number bacteria is represented as relative to the number of inoculated bacteria. N=8 biological replicates per group; *p<0.01 and error bars represent ± standard deviation.

The number of live bacteria that invaded the organoids was calculated as relative to the number of inoculated bacteria within each independent experiment, i.e. the number of invaded bacteria compared to its input inoculum. The treatment of organoids with 0.5 or 0.25 mg/mL OA+EO resulted in a significantly lower number of invaded bacteria (**Figure 1**) compared to the untreated organoids, suggesting that OA+EO has a minor effect on the bacteria while having a major effect on the intestinal organoids.

### 3.2 Altered innate immune gene expression in the intestinal organoids after treatment with OA+EO

**Figure 2:**
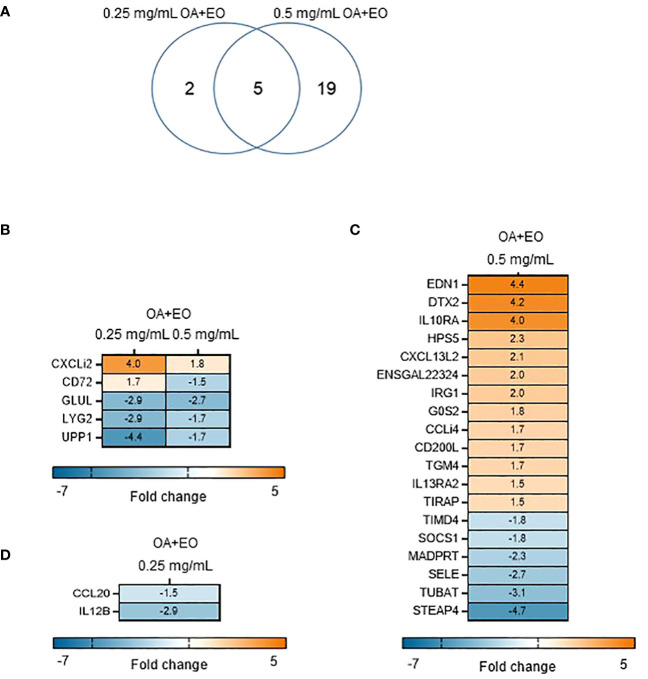
Effect of OA+EO on intestinal organoids. **(A)** The number of significantly differentially expressed genes (DEGs) in organoids treated for 2 days with 0.5 or 0.25 mg/mL OA+EO compared with untreated control organoids. Heat maps illustrate the fold change associated with significant DEGs with a fold change ≥1.5 at p<0.05 that are shared **(B)** or uniquely expressed or uniquely expressed in the high **(C)** or low **(D)** treatment groups. Fold change values are represented on a divergent, intensity color. N=8 biological repeats per group.

To determine the effects the OA+EO on the immune responses of organoids, a high throughput qPCR array was used to analyze the mRNA expression levels of 88 innate immune genes. The organoids were treated for 48 h with 0.5 or 0.25 mg/mL OA+EO and compared to untreated (0 mg/mL) organoids. The number of significantly differentially expressed genes (DEGs) with a fold change (FC) ≥1.5 at p<0.05, was higher after treatment with 0.5 mg/mL compared to 0.25 mg/mL, 24 and 7 respectively, with 5 genes in common (**Figure 2A**).

The higher dose of OA+EO upregulated 16 genes and downregulated 10 genes, whereas the lower dose of OA+EO upregulated one gene and downregulated six genes compared to untreated samples (**Figure 2B**). Five genes were regulated by both concentrations of OA+EO of which one gene is upregulated, the chemokine ligand *CXCLi2* which is an orthologue of human *CXCL8*. *GLUL* (Glutamate-Ammonia Ligase), *LYG2*, and *UPP1* were all downregulated. *UPP1* or Uridine Phosphorylase 1 in human studies was profoundly associated with immune and inflammatory response and correlated with MHC-II and LCK, and is expressed in macrophages and distal enterocytes (28). Lysozyme G like 2 (LYG2) is an antibacterial peptide expressed by heterophils (29). The C-type lectin superfamily member *CD72* was affected in a dose dependent manner, with a higher concentration of OA+EO upregulated CD72 whereas the lower dose downregulated CD72 albeit at a low level (FC -1.8). CD72 is involved in B cell activation and signaling and the cytoplasmic domain contains two immunoreceptor tyrosine-based inhibitory motifs (ITIM1 and 2; 30). In mice, CD72 was shown to be an inhibitory receptor on NK cells regulating cytokine production (31).

Next, we analysed the FC levels of the genes specifically regulated by the different doses of OA+EO. Treatment with the higher dose of 0.5 mg/mL resulted in the upregulation of three genes with an FC >3, *EDN1*, *DTX2* and *IL10RA*. Although Endothelin 1 (*EDN1*) is associated with endothelial cells and vasoconstriction, but recent human single-cell analysis showed high RNA expression in enteroendocrine cells and enterocytes (The Human Protein Atlas, version 23.0). *DTX2* encodes the enzyme Deltex E3 ubiquitin ligase 2 and regulates Notch signaling, a signaling pathway involved in cell-cell communications that regulate a broad spectrum of cell-fate determinations, controlling the homeostasis of occludin and ER stress (32). Only two genes, *CCL20* (FC -1.5) and *IL12*β (FC -2.9), were specially downregulated by a low dose of OA+EO treated enteroids (**Figure 2D**). In summary, treatment of intestinal organoids with a high dose of OA+EO resulted in the activation of innate immune responses, whereas the low dose of OA+EO mostly downregulated genes, while only *CXCLi2* was slightly upregulated by both concentrations of OA+EO.

### 3.3 Treatment of organoids with OA+EO alters inflammation during *Salmonella* challenge

**Figure 3:**
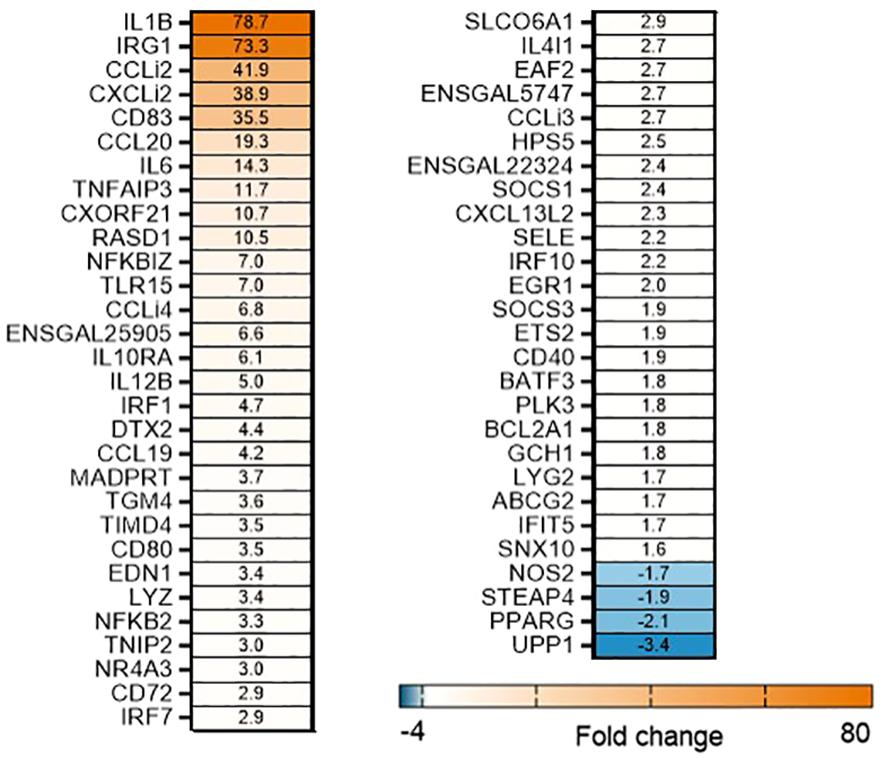
STm upregulates of pro-inflammatory responses in untreated 3D organoids. Heat maps illustrate the fold change associated with significant DEGs in organoids 3 h after challenge with STm compared with control organoids. Significant DEGs with a fold change ≥1.5 at p<0.05 that are represented on a divergent, intensity color. N=8 biological repeats per group.

To determine the effects of OA+EO on altering the innate-immune responses to STm infection in chicken 3D enteroids, we first determined the induction of inflammatory responses to STm without treatment. The infection of organoids with STm resulted in a strong upregulation of (pro)-inflammatory genes at 3 hpi (**Figure 3**). Especially the cytokine (*IL1B*, *IL6*) and chemokine genes (*CCLi2*, *CXCLi2*, *CCL20*) were upregulated with a fold change of 14–79 compared to uninfected controls. A molecular pathway promoting cell activation is the nuclear factor-κB (NF-κB) signaling pathway and STm upregulated several signaling genes such *NFKB2*, *NFKBIZ* and *TNFAIP3* a gene involved in tightly regulating NF-κB activity (33).

**Figure 4:**
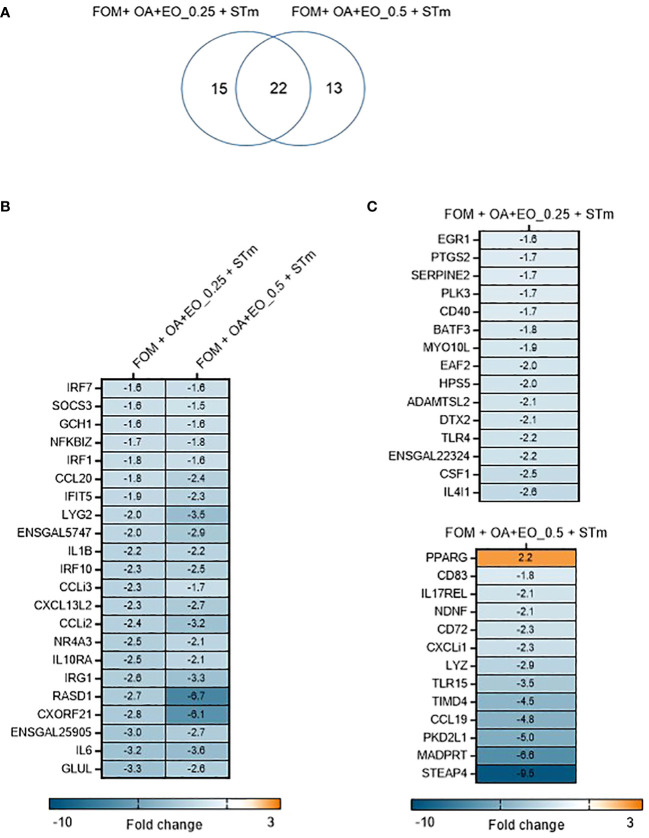
Effect of OA+EO on innate responses of intestinal organoids to STm challenge. **(A)** The number of significantly differentially expressed genes (DEGs) in organoids treated for 2 days with 0.5 or 0.25 mg/mL OA+EO compared and challenged with STm compared with untreated control organoids. Heat maps illustrate the fold change associated with significant DEGs with a fold change ≥1.5 at p<0.05 that are shared **(B)** or uniquely expressed **(C)** in the two treatment groups. Fold change values are represented on a divergent, intensity color. N=8 biological repeats per group.

In contrast, when the organoids were treated with OA+EO and then infected with STm the inflammatory responses were prevented (**Figure 4**). A total of 50 significant DEGs were found after OA+EO treatment and STm challenge, 15 DEGs were regulated by the lower dose and 13 DEGs by the higher dose while 22 DEGs were in common between the high and low doses (**Figure 4**). However, compared to the expression in the STm challenge control group, all DEGs were downregulated after OA+EO treatment of the organoids, with the exception of *PPARG* in the high dose group.

Another striking difference between treatment and bacterial challenge versus bacterial challenge only was the level of expression of DEGs. After STm infection 19 DEGs had a fold change of >4 or <4 and a maximum fold change of 78. In contrast, the OA+EO treated and challenged organoids had 7 DEGs with a fold change of >4 or <4 and a maximum FC of -9 (**Figures 4B, C**). In conclusion, treatment of organoids with OA+EO downregulated the inflammatory responses induced by STm infection aiming to balance homeostatic status.

### 3.4 OA+EO alters the immunometabolic phenotype of organoids during *Salmonella* challenge

**Figure 5:**
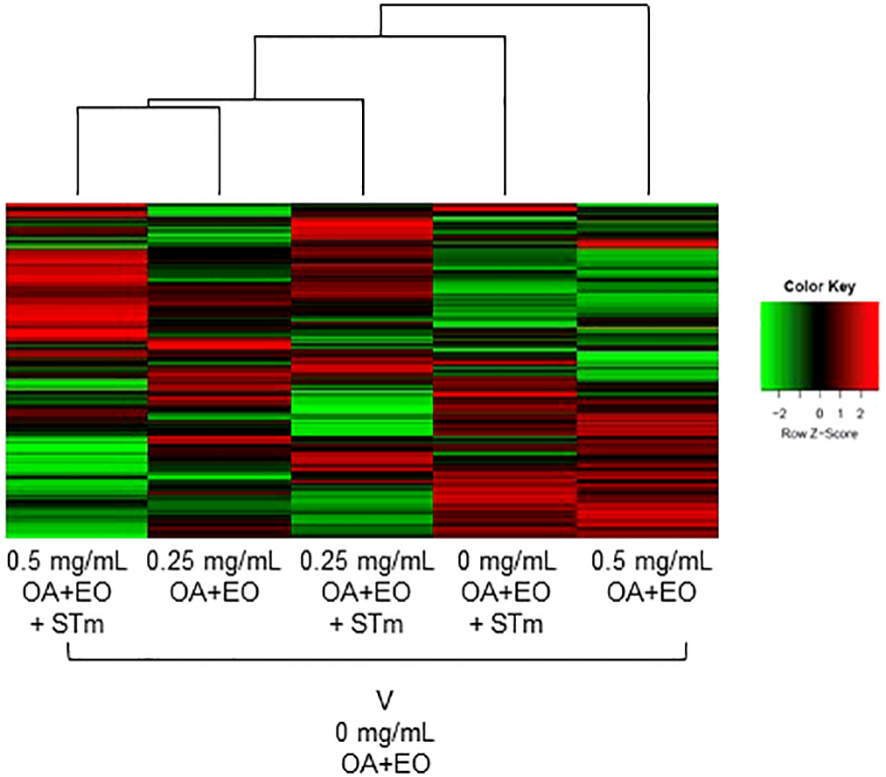
Heat map representing the fold change comparison of peptide phosphorylation on all the five samples compared to the negative control. Red indicates the peptides with increased fold change corresponding to increased phosphorylation and the green indicates the peptides with the decreased fold change corresponding to decreased phosphorylation. The lines connecting each group and the height of those lines represent differences between them. N=6 biological repeats per group.

The kinome array was used to study immune signal transduction pathways occurring in 3D organoids after treatment with high or low dose OA+EO and STm challenge. All the samples were compared with the uninfected and untreated control (0 mg/mL) using the Platform for Integrated, Intelligent Kinome Analysis 2 (PIIKA2) online software (21). The heatmap of phosphorylation changes and experimental group clustering data are shown in **Figure 5**. We observed that high dose OA+EO+STm shows tighter clustering with the low dose OA+EO while these groups do not cluster with high dose OA+EO compared to the untreated control. Low dose OA+EO+STm shows tighter clustering with low dose OA+EO and does not cluster with the high dose OA+EO. These results show that the addition of OA+EO has a significant effect on the signaling of the organoids in the context of the *Salmonella* challenge, especially at the high dose.

**Figure 6:**
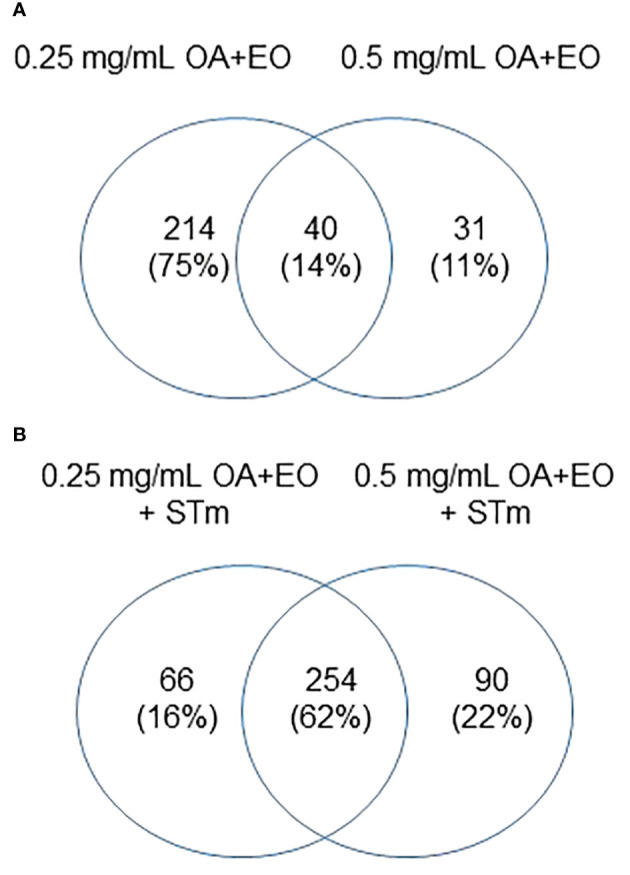
Effects of OA+EO on protein phosphorylation in uninfected and STm infected enteroids **(A)** Significant peptides, illustrated in heat maps, and the shared or unique peptides annotated in Venn diagrams having P-values < 0.05. N=6 biological repeats per group. **(B)** Number of unique proteins statistically significantly differentially phosphorylated in each group and the overlap between groups. All comparisons were carried out against the untreated, uninfected controls, N=6 biological repeats per group, cut off= 0.05.

Similar to the gene expression data, we compared the proteins that were significantly altered by the high dose and low dose OA+EO compared to the control (**Figure 6A**). While the gene expression data showed that most of the changes that occurred were unique to the high dose (**Figure 2A**), the kinome data showed that most of the changes at the protein phosphorylation level (75%) occurred uniquely in the low dose OA+EO group (**Figure 6A**). Most of the changes detected by the peptide array showed a relative decrease in phosphorylation of the proteins unique to the low dose OA+EO while in those proteins unique to the high dose OA+EO, the changes were an increase in phosphorylation (**Supplementary Table**). These results are similar to the gene expression data, where the high dose induced increased gene expression and the low dose mostly reduced gene expression (**Figure 2**). When we considered the *Salmonella* inoculated groups treated with high or low dose OA+EO the majority of the differential protein phosphorylation was shared between the two groups at 62% (**Figure 6B**). The trend in the OA+EO+STm groups was that much of the phosphorylation change relative to control was decreased phosphorylation (**Supplementary Table**).

The pathway overrepresentation analysis of the data generated a list of pathways for each experimental group relative to the control. For all groups, the PI3K-Akt pathway was highly represented in the data (**Tables 1**–**5**). The PI3K-Akt pathway is a central signaling pathway that leads to a number of immune and metabolic responses. Though this pathway is overrepresented in all groups, there are distinct differences in the number of peptides displaying differential phosphorylation and the direction of that phosphorylation (increased or decreased relative to control) (**Supplementary Table**). The *Salmonella* challenge group showed a relatively small number of phosphorylation changes in PI3K-Akt thought many of those were increased phosphorylation, among those several proinflammatory and proliferative response proteins including, Raptor, IRS1, HSP90, FGFR2, ERK, Raf1 (**Supplementary Table**). Interestingly, the high dose OA+EO treated group showed a similar profile to the infection group, with a limited number of phosphorylation changes but among those pro-inflammatory and proliferative (for example NFkB, mTOR, IRS1, HSP90). This contrasts with the low dose OA+EO treated group, which showed a larger number of changes in the PI3K-Akt pathway members, but these were predominantly decreased in phosphorylation, especially amongst the proinflammatory proteins (**Supplementary Table**). Both low and high dose OA+EO+STm showed a relatively large number of changes in the PI3K-Akt pathway, again predominantly as decreased phosphorylation. Therefore, while there was a split in the number of peptides affected and directionally of change between the low and high dose OA+EO, the effects of these two doses merged when in the context of *Salmonella*.

**Table 1:** The 20 significant signal transduction pathways generated by incorporating the statistically significant proteins obtained using the PIIKA2 software followed by normalization between STm infected 3D organoids and negative control.

#Term ID	Term Description	Observed protein count	Background protein count	Strength	False Discovery Rate
hsa04910	**Insulin signaling pathway**	20	133	1.37	2.16E-18
hsa04010	MAPK signaling pathway	23	288	1.1	4.86E-16
hsa04151	**PI3K-Akt signaling pathway**	21	350	0.97	1.9E-12
hsa04660	**T cell receptor signaling pathway**	14	101	1.34	1.9E-12
hsa05206	MicroRNAs in cancer	16	160	1.19	1.9E-12
hsa04931	Insulin resistance	14	107	1.31	1.96E-12
hsa04152	AMPK signaling pathway	14	120	1.26	7.08E-12
hsa05135	Yersinia infection	14	125	1.24	1.03E-11
hsa05200	Pathways in cancer	23	517	0.84	1.58E-11
hsa04012	ErbB signaling pathway	12	83	1.35	2.94E-11
hsa04722	Neurotrophin signaling pathway	13	114	1.25	4.54E-11
hsa05235	PD-L1 expression and PD-1 checkpoint pathway in cancer	12	88	1.33	4.59E-11
hsa04932	Non-alcoholic fatty liver disease	14	148	1.17	5.31E-11
hsa04014	Ras signaling pathway	16	226	1.04	7.51E-11
hsa04068	**FoxO signaling pathway**	13	127	1.2	1.17E-10
hsa05205	Proteoglycans in cancer	15	196	1.08	1.17E-10
hsa04922	Glucagon signaling pathway	12	101	1.27	1.42E-10
hsa01521	EGFR tyrosine kinase inhibitor resistance	11	78	1.34	1.94E-10
hsa04211	Longevity regulating pathway	11	87	1.3	5.36E-10
hsa04380	Osteoclast differentiation	12	122	1.19	9.22E-10

The four pathways highlighted in different colors were further analysed for individual phosphorylation changes (N=6).

**Table 2:** The 20 significant signal transduction pathways generated by incorporating the statistically significant proteins obtained using the PIIKA2 software followed by normalization between 0.5 mg/mL OA+EO treated organoids and negative control.

#Term ID	Term Description	Observed protein count	Background protein count	Strength	False Discovery Rate
**hsa05200**	Pathways in cancer	69	517	0.9	1.31E-36
**hsa04910**	Insulin signaling pathway	42	133	1.28	1.07E-34
**hsa04010**	MAPK signaling pathway	53	288	1.04	4.14E-34
**hsa05230**	Central carbon metabolism in cancer	33	69	1.46	5.13E-32
**hsa04012**	ErbB signaling pathway	33	83	1.38	5.12E-30
**hsa04722**	Neurotrophin signaling pathway	36	114	1.28	5.4E-30
**hsa04151**	PI3K-Akt signaling pathway	49	350	0.92	7.06E-27
**hsa05206**	MicroRNAs in cancer	37	160	1.14	7.84E-27
**hsa04066**	HIF-1 signaling pathway	32	106	1.26	3.17E-26
**hsa01521**	EGFR tyrosine kinase inhibitor resistance	29	78	1.35	6.62E-26
**hsa05161**	Hepatitis B	36	159	1.13	6.62E-26
**hsa05235**	PD-L1 expression and PD-1 checkpoint pathway in cancer	30	88	1.31	6.62E-26
**hsa05205**	Proteoglycans in cancer	38	196	1.06	2.34E-25
**hsa05167**	Kaposi sarcoma-associated herpesvirus infection	37	187	1.07	5.58E-25
**hsa05163**	Human cytomegalovirus infection	38	218	1.02	5.99E-24
**hsa05135**	Yersinia infection	31	125	1.17	1.9E-23
**hsa04014**	Ras signaling pathway	37	226	0.99	1.62E-22
**hsa04068**	FoxO signaling pathway	30	127	1.15	3.32E-22
**hsa05131**	Shigellosis	36	218	0.99	4.68E-22
**hsa04935**	Growth hormone synthesis, secretion and action	29	118	1.17	6.43E-22

The four pathways highlighted in different colors were further analysed for individual phosphorylation changes (N=6).

**Table 3:** The 20 significant signal transduction pathways generated by incorporating the statistically significant proteins obtained using the PIIKA2 software followed by normalization between 0.25 mg/mL OA+EO treated organoids and negative control.

#Term ID	Term Description	Observed protein count	Background protein count	Strength	False Discovery Rate
**hsa05200**	Pathways in cancer	19	517	1.04	2.5E-12
**hsa04151**	PI3K-Akt signaling pathway	16	350	1.13	9.47E-12
**hsa04910**	Insulin signaling pathway	12	133	1.43	9.47E-12
**hsa05230**	Central carbon metabolism in cancer	10	69	1.63	9.47E-12
**hsa05215**	Prostate cancer	10	96	1.49	1.5E-10
**hsa01521**	EGFR tyrosine kinase inhibitor resistance	9	78	1.53	7.27E-10
**hsa04014**	Ras signaling pathway	12	226	1.2	1.01E-09
**hsa04140**	Autophagy - animal	10	130	1.36	1.55E-09
**hsa04150**	mTOR signaling pathway	10	151	1.29	4.97E-09
**hsa05221**	Acute myeloid leukemia	8	66	1.56	4.97E-09
**hsa05206**	MicroRNAs in cancer	10	160	1.27	7.7E-09
**hsa05214**	Glioma	8	72	1.52	7.7E-09
**hsa04010**	MAPK signaling pathway	12	288	1.09	7.93E-09
**hsa05235**	PD-L1 expression and PD-1 checkpoint pathway in cancer	8	88	1.43	2.65E-08
**hsa05231**	Choline metabolism in cancer	8	96	1.39	4.71E-08
**hsa04072**	Phospholipase D signaling pathway	9	147	1.26	5.29E-08
**hsa04218**	Cellular senescence	9	150	1.25	5.89E-08
**hsa04660**	T cell receptor signaling pathway	8	101	1.37	5.89E-08
**hsa04917**	Prolactin signaling pathway	7	69	1.48	1.13E-07
**hsa04722**	Neurotrophin signaling pathway	8	114	1.32	1.26E-07

The four pathways highlighted in different colors were further analysed for individual phosphorylation changes (N=6).

**Table 4:** The 20 significant signal transduction pathways generated by incorporating the statistically significant proteins obtained using the PIIKA2 software followed by normalization between 0.5 mg/mL OA+EO treated organoids infected with treated STm and negative control.

#Term ID	Term Description	Observed protein count	Background protein count	Strength	False Discovery Rate
**hsa04010**	MAPK signaling pathway	53	288	1.08	1.59E-35
**hsa04151**	PI3K-Akt signaling pathway	55	350	1.01	4.51E-34
**hsa05200**	Pathways in cancer	62	517	0.89	8.77E-33
**hsa05230**	Central carbon metabolism in cancer	32	69	1.48	7.77E-32
**hsa04910**	Insulin signaling pathway	38	133	1.27	1.23E-31
**hsa05206**	MicroRNAs in cancer	37	160	1.18	5.19E-28
**hsa04012**	ErbB signaling pathway	30	83	1.37	2.01E-27
**hsa04014**	Ras signaling pathway	39	226	1.05	1.58E-25
**hsa04722**	Neurotrophin signaling pathway	31	114	1.25	2.56E-25
**hsa04066**	HIF-1 signaling pathway	29	106	1.25	9.36E-24
**hsa04152**	AMPK signaling pathway	30	120	1.21	1.18E-23
**hsa01521**	EGFR tyrosine kinase inhibitor resistance	26	78	1.34	3.63E-23
**hsa05131**	Shigellosis	36	218	1.03	4.01E-23
**hsa04931**	Insulin resistance	28	107	1.23	1.29E-22
**hsa05135**	Yersinia infection	29	125	1.18	3.38E-22
**hsa05161**	Hepatitis B	31	159	1.1	8.19E-22
**hsa05205**	Proteoglycans in cancer	33	196	1.04	1.69E-21
**hsa05163**	Human cytomegalovirus infection	34	218	1.01	3.09E-21
**hsa05235**	PD-L1 expression and PD-1 checkpoint pathway in cancer	25	88	1.27	4.95E-21
**hsa04922**	Glucagon signaling pathway	26	101	1.22	5.62E-21

The four pathways highlighted in different colors were further analysed for individual phosphorylation changes (N=6).

**Table 5:** The 20 significant signal transduction pathways generated by incorporating the statistically significant proteins obtained using the PIIKA2 software followed by normalization between 0.25 mg/mL OA+EO treated organoids infected with treated STm and negative control.

#Term ID	Term Description	Observed protein count	Background protein count	Strength	False Discovery Rate
**hsa04010**	MAPK signaling pathway	42	288	1.08	3.34E-28
**hsa05200**	Pathways in cancer	48	517	0.88	8.26E-25
**hsa04151**	PI3K-Akt signaling pathway	40	350	0.97	1.41E-23
**hsa05230**	Central carbon metabolism in cancer	23	69	1.44	2.92E-22
**hsa04910**	Insulin signaling pathway	27	133	1.22	1.73E-21
**hsa05206**	MicroRNAs in cancer	27	160	1.14	1.04E-19
**hsa04012**	ErbB signaling pathway	21	83	1.32	2.16E-18
**hsa04722**	Neurotrophin signaling pathway	23	114	1.22	2.46E-18
**hsa05205**	Proteoglycans in cancer	27	196	1.05	7.73E-18
**hsa04014**	Ras signaling pathway	28	226	1.01	1.84E-17
**hsa04152**	AMPK signaling pathway	22	120	1.18	7.19E-17
**hsa04660**	T cell receptor signaling pathway	20	101	1.21	6.47E-16
**hsa05131**	Shigellosis	26	218	0.99	6.47E-16
**hsa05235**	PD-L1 expression and PD-1 checkpoint pathway in cancer	19	88	1.25	8.81E-16
**hsa05135**	Yersinia infection	21	125	1.14	1.54E-15
**hsa05132**	Salmonella infection	25	209	0.99	2.06E-15
**hsa05167**	Kaposi sarcoma-associated herpesvirus infection	24	187	1.02	2.06E-15
**hsa05215**	Prostate cancer	19	96	1.21	2.81E-15
**hsa05163**	Human cytomegalovirus infection	25	218	0.97	4.27E-15
**hsa04380**	Osteoclast differentiation	20	122	1.13	9.88E-15

The four pathways highlighted in different colors were further analysed for individual phosphorylation changes (N=6).

## 4 Discussion

The objective of this study was to evaluate if the apical-out chicken 3D intestinal organoids that comprise an epithelial cell layer and a lamina propria can be used as a model to mimic the chicken gut responses to feed additives. Evidence of efficacy of feed additives is primarily provided by *in vivo* feeding trials but an *in vitro* model may reduce the number of bird performance studies by way of preselection of compounds. Thereby the global trend to reduce the use of experimental animals will also be addressed.

In this study, treatment of organoids with OA+EO affected the intestinal organoids in a dose-dependent matter. Low dose OA+EO had a moderating effect while the high dose at 0.5 mg/mL appeared more stimulatory based on the number of upregulated genes and the number of proteins being phosphorylated (**Figure 2D**; **Supplementary Table**).

The treatment of chicken intestinal organoids with OA+EO at high and low doses significantly reduced the invasion of *Salmonella*. Although we did not address the mode of action linked to OA+EO in our study, previous studies suggested multiple effects may occur simultaneously including the modulation of the intestinal microbiota, promotion of nutrient absorption and anti-oxidant effects, but antimicrobial properties have been documented (13–15, 33). In our study, a direct antimicrobial activity on *Salmonella* was found as well as modulation of the intestinal oxidative stress based on alteration of the PI3K/AKT and FoxO signaling pathways. The effect on the microbiome can be excluded due to the lack of a microbiome in the model used in this study.

The *Salmonella* challenge alone induced an innate immune response based on mRNA expression of 88 selected genes. The bacteria are recognized by a variety of pathogen pattern recognition receptors leading to the upregulation of proinflammatory cytokines and chemokines including *IL1B*, *CCLi2*, *CXCLi2*, *CCL20*, and *IL6*. Intestinal inflammation seen *in vivo* after *Salmonella* challenge elicits alterations in tissue metabolism and energy-demanding processes such as phagocytosis and generation of oxidative burst (34). *Salmonella* infection alone did not elicit an exceptionally strong response in the organoids as measured by phosphorylation, most likely due to the short period of infection (3 hours). However, this timeframe was sufficient to activate the MAPK, PI3K-Akt, T cell receptor and AMPK signaling pathways (**Table 1**). The induction of pro-inflammatory gene expression and the activation of these signaling pathways have been described *in vivo* after infection with *Salmonella* spp (35, 36). While here we focused on the description of PI3K-Akt signaling alterations by the *Salmonella* inoculation and treatment of the organoids with OA+EO, this central immunometabolic pathway links to the others previously described (37). Our previous work *in vivo* showed pathway alterations in the chicken gut due to *Salmonella* infection in the pathways listed above (35, 38, 39). Our results in this study are consistent with what has been observed *in vivo* in the chicken intestine, providing strong support for the concept of chicken organoids as a model for chicken gut in future mode of action studies.

The two-day treatment of intestinal organoids with OA+EO significantly moderated the response to *Salmonella* challenge both metabolically and immunologically. The inflammatory responses were mostly absent and signaling pathways maintained a more baseline level. In addition, both data sets showed that when comparing the two doses OA+EO in the context of *Salmonella* exposure, the majority of the changes were shared (**Figure 4A** and **Tables 1**–**5**). The changes elicited by the OA+EO with *Salmonella* were decreased gene expression (**Figures 4B, C**) and phosphorylation (**Supplementary Table**) again showing agreement between the gene expression and phosphorylation data sets. Lower inflammatory responses are associated with higher intestinal barrier integrity and lower enteric epithelial leakage. Broilers fed diets with OA+EO and challenged with *Eimeria* spp. and *Clostridium perfringens* showed improved intestinal barrier integrity based on FITC-dextran leakage and increased gene expression of claudin-1 and occludin (11). Other *in vivo* studies have shown increased villus height and villus height to crypt depth ratio in the jejunum of broilers fed diets supplemented with OA (12, 40, 41). In addition, our study suggests that the beneficial effects of OA+EO may be associated with the reduction of the inflammatory status of the intestine because non-typhoidal *Salmonella* serotypes survive and even thrive in the inflamed intestine.

Alternative methods to assess new potential feed additives for poultry are very limited. We previously developed 2D chicken organoids, grown on Matrigel-coated transwell, that can be used to measure transepithelial electrical resistance and demonstrated the beneficial effect of sodium butyrate on epithelial barrier integrity (42). Primary chicken epithelial cell cultures have also been used to demonstrate the inhibitory effect of carvacrol on *Campylobacter* adhesion (43, 44). In this study, we demonstrated that 3D intestinal chicken organoids are a useful alternative method. We demonstrated that treatment of the 3D intestinal organoids with a blend of OA+EO followed by *Salmonella* infection resulted in significantly lower invasion of bacteria and maintenance of homeostatic status instead of an inflammatory response seen after *Salmonella* infection only. The innate immune gene expression and the protein phosphorylation data not only supported each other but also resembled the *in vivo* feeding trials and *Salmonella* challenge.

## Data availability statement

The original contributions presented in the study are included in the article/**Supplementary Material**. Further inquiries can be directed to the corresponding author.

## Ethics statement

The animal study was approved by Roslin Institute Animal Welfare and Ethical Review Body. The study was conducted in accordance with the local legislation and institutional requirements.

## Author contributions

JM: Writing – review & editing, Formal analysis, Investigation. KS: Formal analysis, Investigation, Writing – review & editing, Visualization. JE: Formal analysis, Investigation, Visualization, Writing – review & editing. DB: Formal analysis, Investigation, Visualization, Writing – review & editing. FP: Formal analysis, Investigation, Writing – review & editing. LL: Conceptualization, Funding acquisition, Project administration, Resources, Writing – review & editing. ES: Conceptualization, Project administration, Resources, Writing – review & editing. RA: Formal analysis, Investigation, Methodology, Supervision, Writing – review & editing, Writing - original draft. LV: Conceptualization, Data curation, Funding acquisition, Project administration, Supervision, Writing – original draft, Writing – review & editing.
