# Supplementary material for: Chicken intestinal organoids: a novel method to measure the mode of action of feed additives
Source: Front Immunol. 2024 May 21;15:1368545. doi: 10.3389/fimmu.2024.1368545 (PMC11148291; doi:10.3389/fimmu.2024.1368545)
Supplement: Supplementary file 2 [file Presentation_1.pptx]

## Slide 1
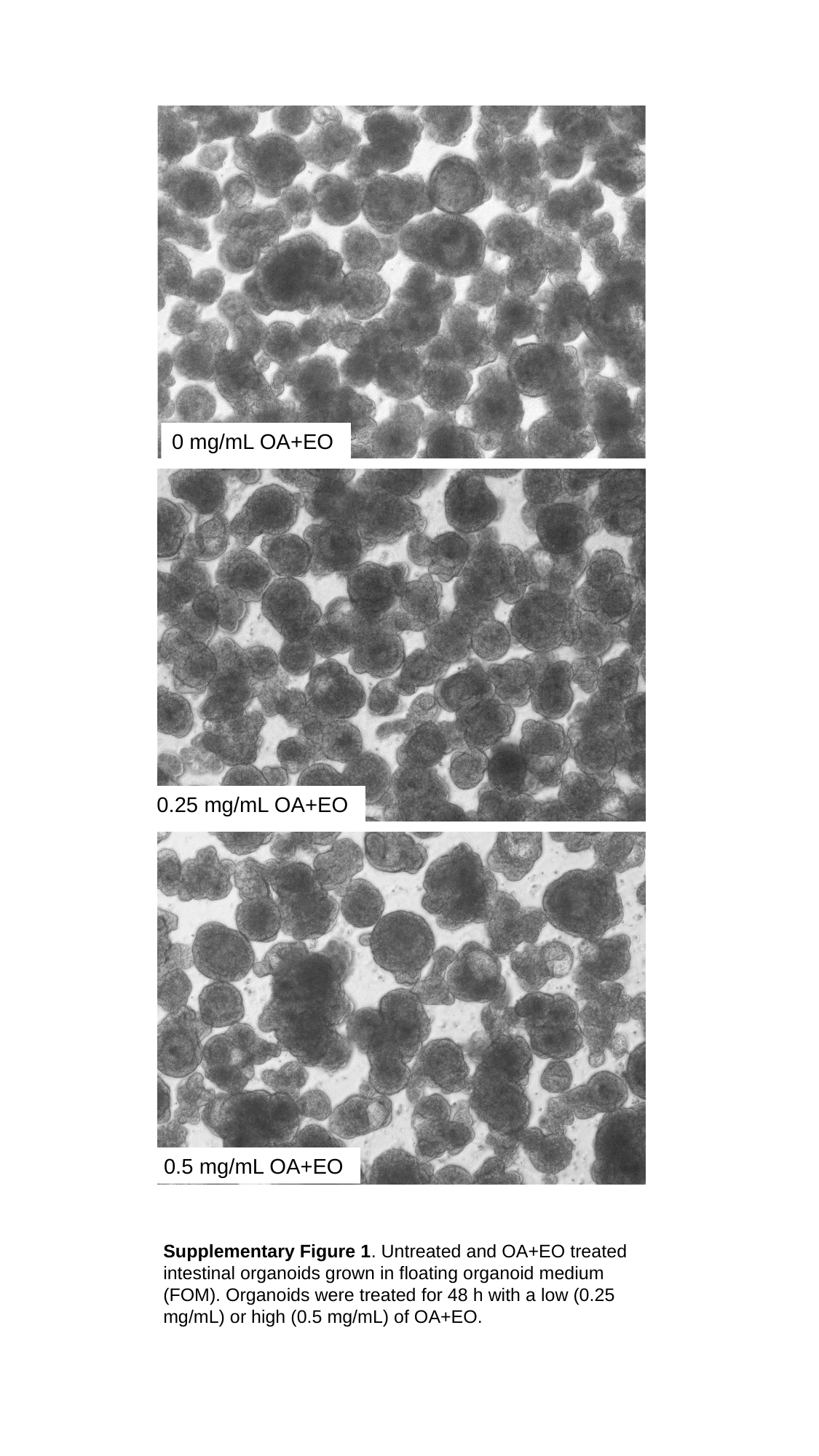

0.25 mg/mL OA+EO
0.5 mg/mL OA+EO
0 mg/mL OA+EO
Supplementary Figure 1. Untreated and OA+EO treated intestinal organoids grown in floating organoid medium (FOM). Organoids were treated for 48 h with a low (0.25 mg/mL) or high (0.5 mg/mL) of OA+EO.
